# The Performance of a Time-Varying Filter Time Under Stable Conditions over Mountainous Terrain

**DOI:** 10.1007/s10546-023-00824-y

**Published:** 2023-07-29

**Authors:** Manuela Lehner, Mathias W. Rotach

**Affiliations:** Department of Atmospheric and Cryospheric Sciences, University of Innsbruck, Innrain 52f, 6020 Innsbruck, Austria

**Keywords:** Complex terrain, Eddy-covariance data processing, Filter time scale, i-Box, Spectral analysis

## Abstract

Eddy-covariance data from five stations in the Inn Valley, Austria, are analyzed for stable conditions to determine the gap scale that separates turbulent from large-scale, non-turbulent motions. The gap scale is identified from (co)spectra calculated from different variables using both Fourier analysis and multi-resolution flux decomposition. A correlation is found between the gap scale and the mean wind speed and stability parameter *z*/*L* that is used to determine a time-varying filter time, whose performance in separating turbulent and non-turbulent motions is compared to the performance of constant filter times between 0.5 and 30 min. The impact of applying different filter times on the turbulence statistics depends on the parameter and location, with a comparatively smaller impact on the variance of the vertical wind component than on the horizontal components and the turbulent fluxes. Results indicate that a time-varying filter time based on a multi-variable fit taking both mean wind speed and stability into account and a constant filter time of 2–3 min perform best in that they remove most of the non-turbulent motions while at the same time capturing most of the turbulence. For the studied sites and conditions, a time-varying filter time does not outperform a well chosen constant filter time because of relatively small variations in the filter time predicted by the correlation with mean flow parameters.

## Introduction

The analysis and description of turbulent motions using eddy-covariance measurements are based on the decomposition of turbulent variables into a time average and fluctuations about this mean, which represent the turbulent motions (Wyngaard 2010). Isolating the turbulent motions by defining an appropriate filter to remove the larger-scale, non-turbulent motions from the raw time series is thus a crucial step in processing eddy-covariance data. The separation of turbulent and non-turbulent scales is in particular important when studying turbulence characteristics, such as similarity relationships since non-turbulent motions cannot be described by the same similarity functions and thus add scatter to the data (Vickers and Mahrt 2003). For other research questions, however, such as the surface-energy balance closure or ecosystem respiration, the total surface fluxes resulting from both turbulent and non-turbulent motions are relevant (Acevedo et al. 2006) so that longer filter times may lead to a better surface-energy balance closure (Mauder and Foken 2006). Here the focus is on the first type of application, for which a clear separation between turbulent and non-turbulent motions is desirable.

Commonly used types of filters include block averaging, also known as mean removal, linear detrending, and other high-pass filters (Aubinet et al. 2012; Donateo et al. 2017; De Franceschi and Zardi 2003; Falocchi et al. 2018). Linear detrending and high-pass filters are oftentimes used to remove trends within individual averaging periods before calculating turbulence statistics. This step is omitted when applying block averaging, so that $\tau _f=\tau _a$, where $\tau _f$ is the filter time and $\tau _a$ is the averaging period. Depending on the application and atmospheric conditions, the different detrending methods and corresponding time constants for high-pass filters have been shown to lead to significant errors in the calculated fluxes (Culf 2000; Donateo et al. 2017; Rannik and Vesala 1999).

Independent of the filter method, an appropriate $\tau _f$ needs to be defined that separates turbulent and non-turbulent motions. Under ideal conditions, a distinct gap occurs in the energy spectra of any turbulent variable that clearly separates turbulent scales from the larger scales and thus defines the appropriate time scale separating the different contributions (Stull 1988). However, particularly under stable conditions, so-called submeso motions may be superimposed on the turbulence, with similar spatial and temporal scales. Submeso motions are defined as non-turbulent motions that are specific to stable stratification and have a spatial scale of about 2 km or less (Mahrt 2014) or a time scale of 1–30 min (Vercauteren and Klein 2015). They include, for example, internal gravity waves (Sun et al. 2015a, b), meandering flow (Cava et al. 2019; Mortarini and Anfossi 2015; Mortarini et al. 2016, 2019; Stefanello et al. 2020), and microfronts (Mahrt 2019; Pfister et al. 2021a, b). In addition, these motions also interact with each other (Cava et al. 2019) as well as with turbulence (Sun et al. 2015a, b; Vercauteren and Klein 2015; Vercauteren et al. 2019a), generating intermittent turbulence bursts (Mortarini et al. 2018; Sun et al. 2002, 2012) and impacting scalar fluxes and the surface-energy balance (Stefanello et al. 2020). Separating these submeso motions from turbulence and thus identifying an appropriate $\tau _f$ can be difficult. For example, spectra calculated by Acevedo et al. (2006) for nighttime periods showed a gap in the co-spectra only if the nights were not characterized by intermittent turbulence.

Different forms of spectral analysis have been used in the past to determine both $\tau _f$ and $\tau _a$ for varying conditions. One common method is based on the Ogive function, which is defined as the cumulative distribution function of the spectral densities (Desjardins et al. 1989; Metzger and Holmes 2008; Oncley et al. 1996). As the contributions to the cumulative distribution and thus the respective (co)variance become increasingly small in the energy gap, the Ogive functions are expected to level off at this location and can thus be used to determine the energy gap scale $\tau _g$. For example, Metzger and Holmes (2008) used a constant threshold of 99.5% of the total covariance to determine $\tau _g$ from Ogive functions of the heat flux under convective conditions, while de Roode et al. (2004), Zhou et al. (2014), and Kang (2020) used a threshold of 2/3 to determine the location of the spectral peak in the spatial spectra from large-eddy simulations. Babić et al. (2012) calculated Ogive functions from the cospectra of the kinematic heat flux and momentum flux for a single 4-h nighttime period with intermittent turbulence, but, since the curves did not converge towards a constant value, the authors concluded that defining a filter time $\tau _f$ based on the Ogive functions is not easy. To estimate turbulent fluxes without the need of explicitly specifying a filter and averaging time, Sievers et al. (2015) fitted a model function to distributions of Ogives that were calculated from a dataset using different filter times and window sizes.

Multi-resolution flux decomposition (MRD) yields similar information as Fourier spectra, that is, the spectral contributions to the total (co)variance, which can be used to identify $\tau _g$ (Howell and Mahrt 1997; Mahrt et al. 2001; Vickers and Mahrt 2003, 2006). Vickers and Mahrt (2003) used MRD to determine $\tau _g$ by identifying the turbulence peak and the location of a subsequent minimum in the spectra or a location at which the spectra level off. This method was further developed by Voronovich and Kiely (2007), who fitted a polynomial to the MRD cospectra, which thus allows an analytical determination of special points, such as maxima and minima, in the fitted function. The latter method was also applied by Babić et al. (2017) to daytime data from the Owens Valley, California, where they found large spatial variations in the identified $\tau _g$ depending on the dominant mesoscale processes. Metzger and Holmes (2008) used the fact that the heat flux often changes sign near the location of the energy gap, as described in Vickers and Mahrt (2003). Analyzing data from unstable conditions and thus assuming a positive sensible heat flux, they identified $\tau _g$ as the period at which the MRD spectra crossed the zero line. A similar approach was also adopted by Kang (2019). Assuming that the heat flux, the moisture flux, and the correlation between temperature and moisture fluctuations have a positive sign above a heated surface, they searched for the scale at which the signs differed from this expectation, finding periods between 9 and 42 min. Wei et al. (2021) and Ren et al. (2019) used yet another type of spectral analysis, specifically a Hilbert-Huang transform, to determine $\tau _g$ during stable conditions.

The energy gap in the spectra occurs typically around a time period of 30–60 min during unstable conditions (Stull 1988; Stiperski et al. 2019). Babić et al. (2017), for example, identified gap scales between 17 and 29 min for different types of daytime thermally driven and channeled valley winds. For this reason, an averaging period $\tau _a$ of 10–60 min is used frequently (Lee et al. 2005), which equals the filter time $\tau _f$ if block averaging is applied. For stable conditions, a shorter $\tau _f$ and $\tau _a$ are commonly used to exclude contributions from non-turbulent submeso motions, with a common $\tau _a$ of 5 min (Cava et al. 2019; Mahrt et al. 1998; Nadeau et al. 2013; Van de Wiel et al. 2003) or 1 min (Banta 2008; Mahrt 2017a, b, 2019; Stiperski et al. 2019). Mahrt and Thomas (2016) even used $\tau _a=$10 s and 6 s for measurements close to the surface under very strong stratification. Donateo et al. (2017) on the other hand, determined a gap scale of about 10 min from temperature spectra and cospectra of the kinematic heat flux for an urban canopy during nighttime.

The appropriate $\tau _f$ thus seems to vary with time and atmospheric conditions. For example, Vickers and Mahrt (2003) determined gap scales between 30 s and 20 min from MRD spectra of a one-month long dataset from CASES99 and also found that they increase with height above the ground. Turbulence peaks can also be seen to move to larger periods for higher measurement levels in the MRD cospectra of Vercauteren et al. (2019b). Stiperski and Calaf (2018), on the other hand, identified distinctly different gap scales for weakly and strongly stable conditions. In addition, the averaging time needed to approach the ensemble average may also depend on the variable. Oncley et al. (1996), for example, identified a $\tau _a$ from the convergence of the Ogives of the momentum flux that was about twice as long as that from the heat flux. Following Lumley and Panofsky (1964), Wyngaard (1973) argued that the averaging time should increase with the order of the statistical moments to reach the same accuracy.

Stationary motions occurring at a time scale longer than $\tau _f$ may positively contribute to total flux estimates (Mahrt 2010, and references therein). The impact of $\tau _f$ on the calculated turbulent fluxes varies, however, strongly among different studies. Kang (2019), for example, compared fluxes calculated with a time-varying filter time $\tau _{fv}$ identified from MRD and fluxes calculated with a constant filter time $\tau _{fc}=30$ min using block averaging and found only negligible differences. Similarly, Feng et al. (2017) calculated block averaged fluxes with averaging periods between 1 and 720 min and found that the fluxes did not differ by more than 3% for $\tau _f<60$ min over a maize field, but that the energy-balance ratio, that is, the ratio between the sum of the turbulent fluxes and the available energy, increases with $\tau _f$. Acevedo et al. (2006), on the other hand, found large differences for $\tau _f$ between 1 min and 30 min for nocturnal conditions with intermittent turbulence, proposing the use of a time-varying filter time. Mahrt et al. (2001) also mention that the impact of $\tau _f$ is more pronounced on variances than on the covariances. Extending $\tau _a$ to values longer than the traditional 30 min may also improve the closure of the surface-energy balance by capturing more low-frequency motions (Finnigan et al. 2003; Foken et al. 2006; Mauder and Foken 2006). Other studies have, however, also found that $\tau _a=30$ min is generally long enough and that longer averaging times do not necessarily lead to a better closure of the surface-energy balance (Charuchittipan et al. 2014). This may, however, differ over tall vegetation (Finnigan et al. 2003).

The goals of this work are to use spectral analysis (i) to identify the gap scale $\tau _g$ for different locations in a steep Alpine valley under stable conditions, (ii) to determine the dependence of the identified $\tau _g$ on the variable, type of spectral analysis, and method used for identification, and (iii) to determine whether a time-varying filter time based on a relation between $\tau _g$ and mean flow characteristics can separate turbulent and non-turbulent motions better than a constant filter time scale. Vickers and Mahrt (2003) have already found a correlation between $\tau _g$ identified from MRD spectra and stability, expressing $\tau _g$ as a function of the Richardson number. Here we use instead a fit between $\tau _g$ and the mean wind speed and the stability parameter *z*/*L* to determine a time-varying filter time. The performance of different constant and time-varying filter times to separate turbulent and non-turbulent motions is evaluated (i) by comparing the scalar-averaged wind speed with the vector-averaged wind speed to determine whether non-turbulent motions are largely removed and (ii) by determining how much of the turbulence range is missed in the spectrum of the vertical velocity. In addition, the dependence of $\tau _g$ on the variable, type of spectral analysis, and method used for identification led us to eventually use an ensemble approach, that is, medians over fits resulting from different (co)spectra and methods. Section 2 briefly describes the measurement sites and instrumentation. The spectra and the identified $\tau _g$ are discussed in Sects. 3 and 4, respectively. Finally, Sect. 5 discusses the impact of $\tau _f$ on the turbulent fluxes and the performance of a time-varying filter time $\tau _{fv}$ compared to a constant filter time $\tau _{fc}$. A summary and conclusions are given in Sect. 6. The different time scales used in this study are summarized in Table 1.

**Table 1:** Definitions of different time scales

Variable	Definition
$\tau _a$	Averaging period
$\tau _f$	Filter time
$\tau _{fc}$	Constant filter time
$\tau _{fv}$	Time-varying filter time
$\tau _g$	Time scale of the energy gap
$\tau _p$	Time scale of the turbulence peak

## i-Box Measurement Sites and Data

**Fig. 1:**
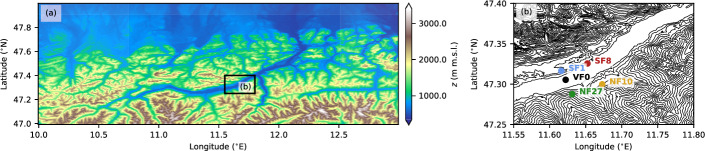
**a** Topography of the Inn Valley, Austria. The black rectangle outlines the location of (**b**). **b** Location of the i-Box measurement sites. Elevation contour lines are at 100-m intervals

Data come from five eddy-covariance stations in the Inn Valley, Austria, which form part of the i-Box (Innsbruck Box, Rotach et al. 2017) measurement installation. The Inn Valley is an approximately southwest–northeast oriented valley in the western part of Austria, which opens north to the Alpine foreland (Fig. 1). The i-Box measurement sites are located about 20 km east of Innsbruck within an approximately 6.5-km long section of the valley. At the location of the i-Box sites, the valley is about 2000 m deep and the valley floor is about 2000 m wide.

The overall goal of the i-Box installation is to collect a long-term dataset of turbulence measurements in a complex Alpine mountain valley (Rotach et al. 2017). The individual sites were thus selected to represent different topographic characteristics (Table 2) and are arranged along two lines across the valley (Fig. 1b). One of the sites, VF0, is located at the almost flat valley floor and is mainly surrounded by grassland and agricultural fields. Two sites are located on the north sidewall, with one site close to the valley floor (SF8) and one site on an almost flat plateau about 200 m above the valley floor (SF1). While SF1 is mainly surrounded by grassland and agricultural fields, SF8 is located at the border between a field and a concrete parking lot and helicopter landing area. Two sites on the south sidewall are located on slopes covered by grassland. The two sites differ mainly in terms of slope angle, with a moderately steep slope of 11$^{\circ }$ (NF10) and a steep slope of 25$^{\circ }$ (NF27). A sixth eddy-covariance tower is located on a nearby mountain top, which is, however, not used in this study because of frequent data gaps.

**Table 2:** Site characteristics, elevation, slope angle, and measurement levels for sonic anemometers and gas analyzers

Site$^\textrm{a}$	Characteristic	Elev. (m)	Slope$^a$ ($^{\circ }$)	*z* (m)—sonic	*z* (m)—gas analyz
VF0	Valley floor	545	0	4.0, 8.7, 16.9	4.0, 16.9
SF8	South-facing	575	9	6.1, 11.2	11.2
SF1	South-facing	829	3	6.8	6.8
NF10	North-facing	930	11	5.7	5.7
NF27	North-facing	1009	25	1.5$^\textrm{b}$, 6.8	6.8

$^\textrm{a}$While the last two digits of the site names refer to the slope angle, these slope angles are based on an earlier assessment and may differ from those given in the table, which are based on an assessment by Lehner et al. (2021). Site names are, however, kept for consistency with previous i-Box publications

$$^\textrm{b}$$Since September 2017

Measurements started in 2012 or 2013, depending on the station. In the present study, data are used from the 7-year period between 2014 and 2020. All five sites are instrumented with CSAT3 sonic anemometers (Campbell Scientific Ltd., Logan, Utah, USA), with three measurement levels between 4 and 17 m a.g.l. (above ground level) at VF0 (Table 2). The other four stations are equipped with a single sonic anemometer at 5.7–6.8 m a.g.l. and an additional CSAT3 at 11.2 m a.g.l. at SF8. A second CSAT3 was also installed at NF27 in September 2017. Fast-response humidity measurements are made at 4.0 and 17 m a.g.l. at VF0 with an open-path, infrared gas analyzer (EC150, Campbell Scientific Ltd.) and until 2020 a Krypton hygrometer (KH20, Campbell Scientific Ltd.), respectively. KH20 hygrometers were operated at one measurement level at each of the other sites initially. In September 2017, the KH20 and respective CSAT3 at NF27 were replaced with the combined Irgason (Campbell Scientific Ltd.). The remaining Krypton hygrometers at VF0, SF8, SF1, and NF10 were replaced with Irgasons in fall 2020. The lowest measurement level at VF0 (4.0 m a.g.l.), SF8 (6.1 m a.g.l.), and NF27 (1.5 m a.g.l.) will be referred to as VF0_lvl1, SF8_lvl1, and NF27_lvl1, respectively. Analogous terminology will be used for the second measurement levels (VF0_lvl2, SF8_lvl2, and NF27_lvl2) and the third level at VF0 (VF0_lvl3). Air temperature and humidity measurements used for flux corrections come from PT100 temperature and HT-1 humidity sensors (HC2A-S, Rotronic, Bassersdorf, Switzerland). Pressure is measured with Setra 278 sensors (Setra Systems, Inc., Boxborough, Massachusetts, USA).

For comparing the impact of different filter time scales on turbulent fluxes, i-Box data are processed using block averaging without an additional high-pass filter, so that $\tau _a$ equals $\tau _f$. Data are processed using averaging periods of 0.5, 1, 2, 3, 5, 10, 15, and 30 min.^1^ Before calculating turbulent statistics, raw 20-Hz data are quality controlled and rotated into a streamline coordinate system using double rotation. During quality control, data are removed if the instrument quality flag is set; if data points are classified as spikes; and if measurements exceed 30 m s$^{-1}$ for the horizontal wind components, 10 m s$^{-1}$ for the vertical wind component, and 50 g m$^3$ for water vapor density or are outside the range $-20-40^{\circ }$C for sonic temperature. Removed data are replaced by random values drawn from a Gaussian distribution with the mean and standard deviation calculated from a 30-s window. If more than 10% of the data within a single averaging interval are replaced, the calculated turbulent statistics are excluded from further analysis. Flux corrections are applied to the (co)variances, including a frequency response correction (Aubinet et al. 2012; Moore 1986) with co(spectral) models following Moore (1986), Højstrup (1981), and Kaimal et al. (1972); a sonic-heat flux correction of the vertical heat flux and temperature variance (Schotanus et al. 1983); a WPL correction of the vertical moisture flux (Webb et al. 1980); and an Oxygen correction of the vertical moisture flux based on measurements from Krypton hygrometers (Van Dijk et al. 2003).

## (Co)spectra

### Calculation of (co)spectra

Two different types of (co)spectra are calculated to determine the gap scale $\tau _g$: Fourier decomposition and subsequent calculation of Ogive functions from the Fourier (co)spectra and MRD. Fourier (co)spectra are calculated using the Welch method (Welch 1967) for 1-h long periods that overlap by 30 min to increase the sample size. Ogive functions are defined as the cumulative distribution function of the spectral densities (Desjardins et al. 1989; Metzger and Holmes 2008; Oncley et al. 1996):

1 $$\begin{aligned} Og_{xy}\left( f_0\right) = \int _\infty ^{f_0} S_{xy}(f)df, \end{aligned}$$

where *f* and $f_0$ are the frequency and $S_{xy}$ is the spectral density of the variables *x* and *y*.

The MRD approach is described in detail in Vickers and Mahrt (2003). To summarize the basic concept, two time series $x_i$ and $y_i$ with $i = 1, 2, \dots 2^M$ data points are split into consecutively smaller blocks by cutting the individual blocks into halves until $n=2^M$ blocks with a length of only 1 data point remain. In each step, the block average is first subtracted before splitting the block into two parts. The cospectrum of variables *x* and *y* is then defined as:

2 $$\begin{aligned} D_{xy}\left( m+1\right) = \frac{1}{2^{M-m}} \sum _{n=1}^{2^{M-m}} {\overline{x}}_n \left( m\right) {\overline{y}}_n \left( m\right) , \end{aligned}$$

where *m* is the scale, with $m=M$ the lowest-order mode corresponding to the mean over the whole time series with $2^M$ data points and $m=0$ the highest-order mode corresponding to the mean over blocks of one data point, and ${\overline{x}}_n$ the average over block *n* of the time series $x_i$. $D_{xy}$ has the same units as the covariance $\overline{x'y'}$. The spectrum of a single variable *x* can be calculated analogously as $D_{xx}$. MRD (co)spectra are computed for overlapping time series of $2^{16}$ data points corresponding to approximately 55 min, with a new time series starting every 30 min.

Only stable (co)spectra with positive *z*/*L* in both 30-min periods of each 1-h period, for which the (co)spectra are calculated, are used to determine the gap scale.

### Binned (co)spectra

#### Turbulent Peak

**Fig. 2:**
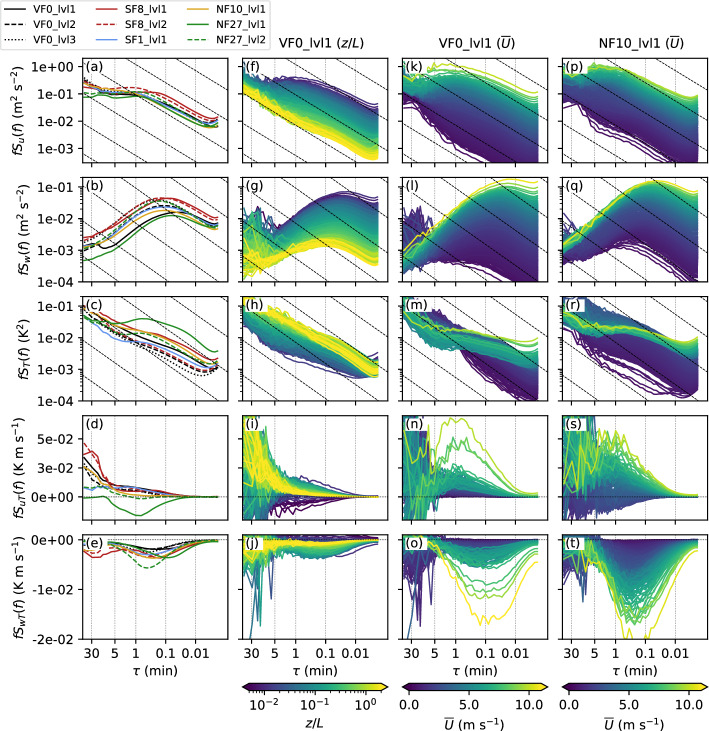
**a**–**e** Mean (co)spectral densities of *u*, *w*, *T*, *uT*, and *wT* calculated over all stable (co)spectra at different sites (line colors, see top-left legend). **f**–**t** (Co)spectral densities at VF0_lvl1 color coded by (**f**–**j**) *z*/*L* and (**k**–**o**) ${\overline{U}}$ and at (**p**–**t**) NF10_lvl1 color coded by ${\overline{U}}$. Mean (co)spectra are shown for each variable-sized bin containing 20 (co)spectra. Line color indicates the mean *z*/*L* or ${\overline{U}}$ for the respective bin. Sloping dashed lines show a $-2/3$ slope

Mean (co)spectra calculated over the selected stable periods are shown in Fig. 2a–f for all five i-Box sites and each vertical measurement level. Overall, all (co)spectra show a more or less pronounced turbulent peak between 0.1 and 1 min. The exact location of the peak differs, however, strongly from site to site and also from variable to variable, with a smaller turbulent-peak time scale $\tau _p$ of about 0.1 min in the vertical velocity compared to the horizontal components with $\tau _p\approx 1$ min (Fig. 2a, b). The difference in the location of the turbulent peak is equally visible between the vertical and horizontal heat fluxes (Fig. 2d, e), as well as in the moisture fluxes (not shown). In the vertical velocity, there is also a shift of the peak with distance from the ground at all three sites with multiple measurement levels (VF0, SF8, and NF27). The size of the largest and most energy-containing eddies is expected to decrease close to the ground. Sun et al. (2020) have shown that under stable conditions the length scale of the peak in the vertical velocity spectra is slightly lower than the measurement height *z*. The height dependence is also reflected in the surface-layer scaling by Kaimal et al. (1972), who have shown that appropriately scaled spectra plotted against a normalized frequency that is proportional to the height *z* collapse onto a single curve in the inertial subrange (Kaimal and Finnigan 1994; Vickers and Mahrt 2003). The focus of this work is on the gap scale and as Vickers and Mahrt (2003) have pointed out, the relation between the peak scale and the gap scale is not known, but they have also shown that the gap scale increases equally with height above ground. The turbulent peak in the spectra of the horizontal wind component is also shifted towards smaller time scales at the lowest level of SF8 compared to the other sites. A possible explanation is the location of the site next to a steep embankment at the border between a concrete area and cropland. While the measurement height of 6.1 m a.g.l. (Table 2) refers to the height above the parking lot south of the mast, the height above ground is lower with respect to the area north of the embankment.

While the mean vertical velocity spectrum decreases continuously with increasing $\tau$ for $\tau >\tau _p$ and $S_w(f)$ approaches zero, $S_{uT}(f)$ (Fig. 2d), $S_T(f)$ (Fig. 2c) and $S_q(f)$ (not shown) show a distinct increase at large scales. The exception is NF27_lvl1, where $S_{uT}$ goes to zero at $\tau \approx 10$ min. This is also the only location with a mean negative $S_{uT}$, indicating an upslope turbulent heat flux, directed against the predominantly downslope oriented flow during nighttime at this location.

#### Dependency on Mean Wind Speed and Stability

To analyze the dependency of the (co)spectra on mean flow parameters, they are binned based on mean 1-h values of *z*/*L* and scalar-averaged wind speed ${\overline{U}}$ calculated from individual 30-min values, where *z* is the measurement height and *L* the Obukhov length (Fig. 2f–t). Binning the (co)spectra based on 1-h averages of data processed with $\tau _{fc}=1$ min does not change the results shown in Fig. 2 significantly (not shown). The (co)spectra are binned into equal-sized bins of 50 (co)spectra per bin, which, however, means that the range of the binning variables *z*/*L* and ${\overline{U}}$ varies among bins. The spectra of the wind components show the expected behavior with lower values of $S_u(f)$ and $S_w(f)$ for small ${\overline{U}}$ (Fig. 2k, l) and large *z*/*L* (Fig. 2f, g). In other words, the variance is smallest for stable and weak-wind conditions, when shear production is small and buoyancy damping large. The turbulence peak in $S_w$ shifts to smaller time scales for higher wind speeds, consistent with the normalized frequency used to scale the spectra in Kaimal et al. (1972), which is inversely proportional to the mean wind speed. In the spectral model by Kaimal et al. (1972), the location of the turbulence peak remains a function of stability, with the peak shifted to lower non-dimensional frequencies for less stable conditions. The shift in the turbulence peak with *z*/*L* is not as clear as for wind speed, but it decreases slightly with increasing stability. High stability is, however, typically associated with low wind speeds, so that the two parameters are not independent of each other.

The impact of stability and wind speed is similar for all periods in the inertial subrange, that is, below approximately 1 min. For larger scales, the spectra decrease for low stabilities and high wind speeds with increasing time. For weak winds and high stability, the spectra continue to increase, suggesting the presence of submeso motions. A similar absence of a spectral gap under very stable conditions and a continuous increase of the spectra of horizontal wind with increasing spatial scales was also found by Vercauteren et al. (2019a) and attributed to the impact of submeso motions.

For temperature, the dependency on *z*/*L* and ${\overline{U}}$ is not as clear as for the wind components. While spectral density increases with increasing wind speed for high frequencies, the highest spectral densities at low frequencies occur with very weak winds (Fig. 2m). This suggests that submeso motions contribute at these scales during low-wind conditions. For stability, the spectral densities of *T* are highest for medium values of *z*/*L* and decrease towards both very stable and near-neutral conditions (Fig. 2h). This is consistent with the explanation given by Mahrt et al. (1998) for the highest sensible heat fluxes occurring for medium *z*/*L* in that temperature fluctuations are small under near-neutral conditions. The observed dependency of the temperature fluctuations on *z*/*L* is equally visible in the cospectral densities of both the vertical and horizontal heat fluxes (Fig. 2i, j).

#### Dependency on the Flow Regime

**Fig. 3:**
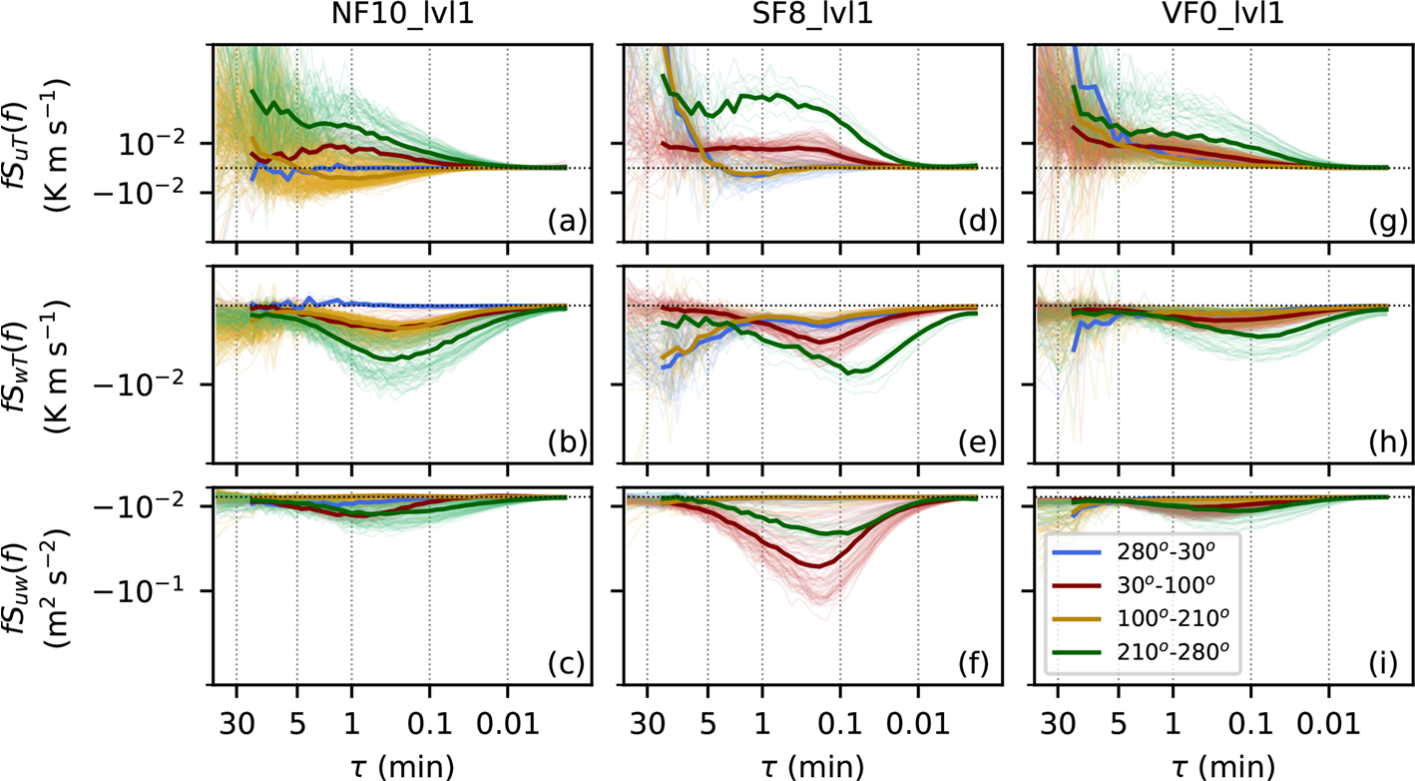
Similar to Fig. 2, showing the mean (co)spectral densities of *uT*, *wT*, and *uw* at **a**–**c** NF10_lvl1, **d**–**f** SF8_lvl1, and **g**–**i** VF0_lvl1 as a function of wind direction. Red and green lines represent the up-valley and down-valley direction, respectively. Yellow lines correspond to the downslope direction at NF10 and the upslope direction at SF8 and blue lines, vice-versa, the upslope direction at NF10 and the downslope direction at SF8. Thick lines show the medians over the respective wind direction ranges and dotted horizontal lines indicate zero for the cospectral densities

In contrast to the valley floor (VF0), the cospectra of *uT* at the slope site NF10 change sign for low ${\overline{U}}$ from generally positive values, that is, a turbulent transport in the direction of the main wind direction, to slightly negative values, that is, a turbulent transport against the main wind direction. This sign change is a result of different flow regimes at the north-facing slope, which becomes apparent when stratifying the data according to wind direction (Fig. 3a–c). The low-wind speed cospectra at NF10_lvl1 with distinctly different behavior are associated with southerly downslope flows (yellow lines in Fig. 3). These katabatic winds are very shallow, which is indicated by the almost zero, but sometimes very weak upward transport of momentum (Fig. 3c), showing that the wind-speed maximum is located near or below the measurement level at 5.7 m a.g.l. The oftentimes negative sign of $S_{uT}(f)$ shows that the turbulent transport advects warmer air from further down the slope against the mean wind direction. Similar differences in the spectral densities and in $S_{uT}$ and $S_{uw}$ for katabatic winds can also be observed at NF27 (not shown). In addition to the distinctly different katabatic flow regime, other regimes can be identified as well at NF10 (Fig. 3a–c). Specifically, the overall largest magnitudes of both $S_{wT}(f)$ and $S_{uT}(f)$ and of $S_{uw}(f)$ occur together with a westerly down-valley direction (green lines), while easterly up-valley flows (red lines) lead to smaller magnitudes.

The effect of different flow regimes on the (co)spectra is also visible at other sites, for example, at VF0 and SF8 (Fig. 3d–i). At SF8, westerly down-valley winds (green lines) are also characterized by larger $S_{uT}(f)$ and $S_{wT}(f)$ than easterly up-valley winds (red lines) and a shift of the turbulent peak to lower and higher $\tau$ in $S_{wT}(f)$ and $S_{uT}(f)$, respectively (Fig. 3e, d). At the same time, $S_{uw}(f)$ is lower for down-valley flows (Fig. 3f) and $S_{vw}(f)$ is positive in contrast to up-valley flows (not shown), which can also be seen at SF1. Thermally driven up-valley wind periods with stable conditions are mainly restricted to the evening and early night, whereas the down-valley winds can extend beyond sunrise, when wind speeds start to increase near the surface (Lehner et al. 2021) and buoyancy starts to contribute positively to turbulence production. The pronounced shift in the turbulent peak of $S_{wT}$ can also be seen in the spectra of the vertical velocity (not shown) and may be related to stronger turbulence anisotropy in the down-valley flows due to submeso motions or terrain effects, with higher terrain just north of the measurement site. During periods of downslope flows (blue lines), cospectral densities are general weak and $S_{uT}$ becomes slightly positive, similar to the downslope flows at NF10 and NF27. Even at VF0, the largest cospectral densities occur for down-valley directions (green lines). Flows with a more southerly direction, on the other hand, include a cross-valley wind component, which could be related to previously documented outflows from tributary valleys to the south (Babić et al. 2021).

In summary, the (co)spectral densities clearly depend on the mean flow parameters, but this dependency is not consistent across the whole frequency range and it varies among the different variables. For example, submeso motions that influence the large-scale end of the (co)spectra, impact the horizontal motions more than the vertical. The distinctly different shapes of the (co)spectra also suggest that not all variables are equally well suited for identifying a filter time because of, for example, the lack of a pronounced gap in the (co)spectra.

## Gap Time Scale

### Identification of the Gap Time Scale from (co)spectral Densities

To identify the gap time scale, the methods by Vickers and Mahrt (2003, VM03 hereafter) and by Voronovich and Kiely (2007, VK07 hereafter) are followed. While Vickers and Mahrt (2003) used only MRD cospectra, we are applying their method not only to the MRD (co)spectra $D_{xy}$, but equally to the Fourier (co)spectra *S*(*f*) and to (co)spectra derived from the Ogive functions $\varDelta Og$. VK07 is similarly applied to all three types of (co)spectra. The only difference is that the MRD (co)spectra are first smoothed with a 1-2-1 filter as in VM03, whereas the other (co)spectra are smoothed with a LOWESS (locally weighted scatterplot smoothing) function. While $\varDelta Og$ are very similar to the original Fourier (co)spectra and can thus be expected to yield similar $\tau _g$, they do differ somewhat as a result of the bin averaging applied to the raw Fourier (co)spectra to reduce the number of frequency values and thus the amount of the data being stored. In the (co)spectra derived from Ogive functions, the sign information is lost as well since they are calculated from normalized Ogives.

**Fig. 4:**
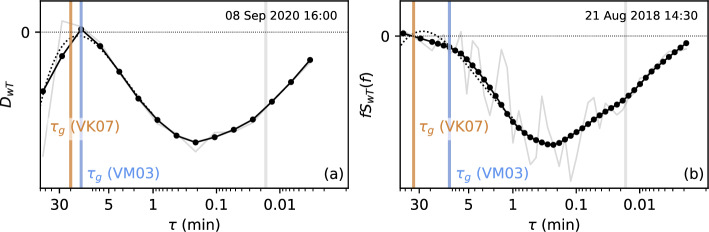
Example **a** $D_{wT}$ and **b** $S_{wT}$ cospectra showing the gap identification. Solid lines with markers show the smoothed cospectra, light solid lines the original unsmoothed cospectra, and the dotted lines the fifth-order polynomial fit used by VK07. Vertical blue and brown lines indicate the gap scales identified using the methods by VM03 and VK07, respectively, and the vertical gray line indicates the lower limit of $\tau =1$ s for identifying the turbulence peak. The example $D_{wT}$ in (**a**) and $S_{wT}$ in (**b**) were selected to visualize the methods best and are not for the same time period

Individual steps of the identification algorithms are visualized for two example cospectra in Fig. 4, specifically for an MRD and a Fourier cospectrum of *wT*. For VM03, the turbulence peak $\tau _p$ is identified in the smoothed (co)spectra as the location where the slope of the (co)spectrum $dS/d\tau$ or $dD/d\tau$ first becomes negative above a threshold value of $\tau =1$ s. If the dominant part of the (co)spectrum in the range $\tau =1-300$ s is negative as in the examples shown in Fig. 4, the sign of the whole (co)spectrum is first changed to ensure that the turbulence peak is a maximum and not a minimum. The (co)spectrum is discarded if no sign change, that is, no turbulence peak is found or if $dS/d\tau$ is already negative at $\tau =1$ s. The gap scale $\tau _g$ is then defined as the location where $dS/d\tau$ changes sign again, that is, at the first minimum in the (co)spectrum, which deviates by at least 5% from the peak value, for example, $\tau _g$ identified from $D_{wT}$ in Fig. 4a (blue line). Figure 2 suggests that many of the (co)spectra do not have a pronounced gap, for example, $S_{wT}$. If no minimum exists, $\tau _g$ is identified as the location where the (co)spectral density stops to contribute significantly to the total (co)variance, for example, $\tau _g$ identified from $S_{wT}$ in Fig. 4b (blue line). This point is defined as the location where the (co)spectrum normalized by the cumulative sum drops below an arbitrary threshold of 0.005, that is, the contribution of the (co)spectral density to the total (co)variance drops to below 0.5%.

For the second method, following VK07, a fifth-order polynomial is fitted to the individual smoothed (co)spectra (dotted lines in Fig. 4) and the curves with a fit error exceeding 0.15 are rejected. The location of turbulence peak $\tau _p$ is then determined analytically as the first extremum in the fitted curve between $\tau =1$ and 1200s. Further extrema, roots, and inflection points are found for $\tau >\tau _p$ and $\tau _g$ is derived from the polynomial fit *p* as the location where $p(\tau _g) = p(\tau _m) + 0.02 \left( p(\tau _p) - p(\tau _m) \right)$, with $\tau _m$ being the minimum $\tau$ of the first extremum, first root, and second inflection point after $\tau _p$. A detailed description of the method can be found in VK07 and in Babić et al. (2017).

### Gap Times Scales from Different (co)spectra and Identification Methods

**Fig. 5:**
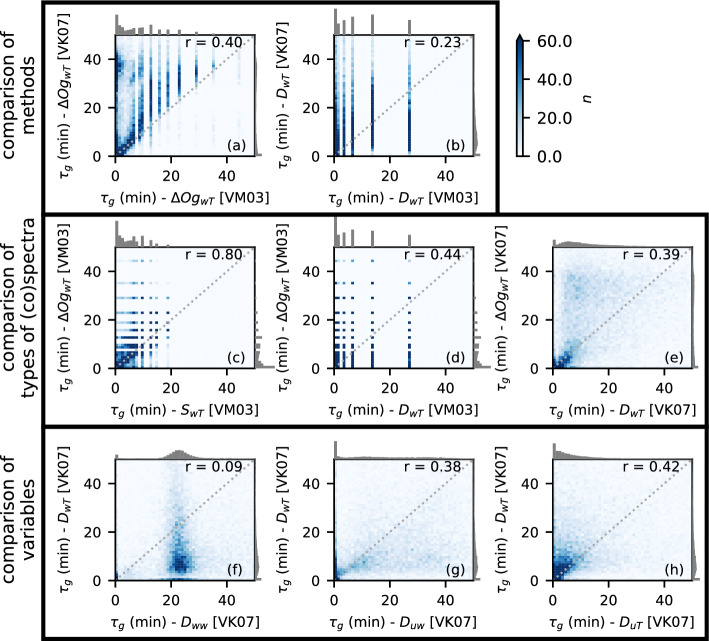
Comparison of $\tau _g$ identified from different (co)spectra and methods at VF0_lvl1: comparison of VM03 and VK07 applied to **a** $\varDelta Og_{wT}$ and **b** $D_{wT}$; comparison of $\tau _g$ **c**, **d** from VM03 applied to $\varDelta Og_{wT}$, $D_{wT}$, and $S_{wT}$ and **e** from VK07 applied to $\varDelta Og_{wT}$ and $D_{wT}$; **f**–**h** comparison of $\tau _g$ from $D_{wT}$ and $D_{ww}$, $D_{uw}$, and $D_{uT}$ using VK07. Gray histograms along the *x*- and *y*-axis show the distributions of $\tau _g$ from the respective method and variable and *r* in the top-right corner of each subfigure is the Spearman correlation coefficient

In this section, the gap time scales identified from the (co)spectra of different variables, different types of (co)spectra, and different identification methods are compared to evaluate the sensitivity of the identified gap scale to these different options. Figure 5a,b compare all $\tau _g$ from VM03 and VK07 identified from $\varDelta Og_{wT}$ and $D_{wT}$. It has to be kept in mind that VK07 determines $\tau _g$ analytically from a fit to the spectra, whereas VM03 identifies the gap in the original spectra, so that the resolution decreases with increasing $\tau$ for VM03 (Fig. 4). Overall most of the data are located above the 1:1 line for $\varDelta Og_{wT}$ (Fig. 5a), indicating larger $\tau _g$ identified by VK07 than by VM03. The reason may be found in the fact that VM03 is applied to the raw (co)spectra, which may contain small minima even in the smoothed curves, which are removed in the polynomial fits used by VK07. This may lead to particularly large differences when applied to MRD (co)spectra with a coarser resolution (Fig. 5b). The agreement between the two methods depends also on the variable (not shown) and the cospectrum of *wT* in Fig. 5a, b was simply chosen as an example.

Fourier (co)spectra are binned into logarithmically spaced frequency bins before outputting them. Since the resolution decreases for lower frequencies and fewer data points are averaged in the low-frequency bins, the (co)spectra are noisier and the application of VM03 does not work well beyond approximately $\tau =30$ min. This means that large $\tau _g$ cannot be identified based on a combination of Fourier (co)spectra and the VM03 method, so that the resulting distributions of $\tau _g$ are biased towards lower values (Fig. 5c). For low values of $\tau _g$, the agreement with $\tau _g$ identified from $\varDelta Og$ is, however, good. The VK07 method is not affected since the polynomial fit can be extended to larger $\tau$. Similar results are found for other variables (not shown).

Since the MRD (co)spectra are determined by splitting the time series into consecutively smaller blocks of data, the resolution of the MRD (co)spectra is much coarser than the resolution of the Fourier and $\varDelta Og$ (co)spectra for low frequencies (Fig. 4). This is also reflected in $\tau _g$ identified by the VM03 method, which, for example, can be only 6.8 or 13.7 min in the range $\tau =5-15$ min (Fig. 5d). Since $\tau _g$ is determined analytically from the fitted polynomial curve in VK07, continuous values of $\tau _g$ are possible for MRD (co)spectra as well (Fig. 5e). Mahrt et al. (2001) mention that the spectral gap identified from Fourier (co)spectra occurs at somewhat larger periods than the gap identified from MRD (co)spectra. With the VM03 method, overall little correlation is found between $\tau _g$ identified from the two types of cospectra (Fig. 5d). With the VK07 method, on the other hand, the values agree very well for $\tau _g$ smaller than about 10 min without a clear bias (Fig 5e). For larger values, the correlation is low, but most of the data points are located above the 1:1 line indicating higher values from the Fourier than from the MRD cospectra.

As seen in the (co)spectra (Fig. 2), the location of the gap varies among the different variables, which means that $\tau _g$ will equally vary depending on the variable used for identification. Figures 5f–h show comparisons of $\tau _g$ identified from $D_{wT}$ using VK07 with $\tau _g$ from different other MRD (co)spectra. Since the spectral densities of *w* decrease continuously after the turbulence peak without a spectral gap (Fig. 2b), the identification of $\tau _g$ mostly fails, yielding an almost constant value of about 25 min (Fig. 5f), mostly independent of the type of spectrum and method used. Identification from spectra of *T* equally fails most of the time despite the presence of a spectral gap, but resulting in very low values close to zero (not shown). Correlations between $\tau _g$ identified from different variables are generally low with large scatter. A relatively high number of data points close to the 1:1 line can be found for $D_{uw}$ (Fig. 5g), $D_{uT}$ (Fig. 5h), and $D_{wq}$ (not shown), particularly for $\tau _g$ smaller than 20 min, but correlations are overall low. While scatter is relatively large, $\tau _g$ from $D_{uw}$ tends to be somewhat larger than from $D_{wT}$, particularly for larger $\tau _g$ (Fig. 5g), and $\tau _g$ from $D_{wT}$ are again slightly higher than from $D_{uT}$ (Fig. 5h). This is likely related to the stronger impact of submeso motions in the high-frequency range of the cospectra of *uT* and *wT* than in the momentum cospectrum. At NF10 and NF27_lvl1, which are both strongly influenced by downslope flows during nighttime, no systematic difference can be seen between $\tau _g$ from $D_{uw}$ and $D_{uT}$ or $D_{wT}$ (not shown). Overall, results clearly depend on the selected variable, but also on the type of (co)spectra and method used to identify $\tau _g$. While some yield clearly erroneous results (for example, *w* spectra), others may differ, but it is not immediately obvious which one of them, or if any is incorrect. Further analysis is thus partly based on an ensemble approach, using all of the methods, types of (co)spectra, and variables excluding only clearly unrealistic results.

### Site Dependency

**Fig. 6:**
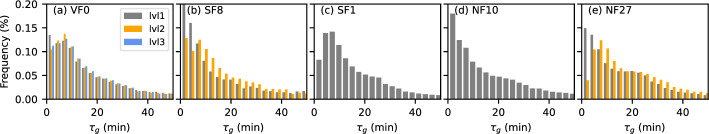
Distributions of $\tau _g$ identified from $D_{wT}$ using VK07 for different sites and measurement levels

Distributions of $\tau _g$ identified from $D_{wT}$ using VK07 are compared for the different measurement sites in Fig. 6. The distributions are overall similar for all sites and vertical levels. This is also true for other variables and types of (co)spectra; the distributions in Fig. 6 are again only an example. The variable, type of (co)spectrum, and method have thus a larger influence on the detected $\tau _g$ than the location. Notable exceptions occur for low $\tau _g$ both at SF8 and NF27. A larger fraction of values are in the lowest two bins at the lower level than at the upper level, that is, small $\tau _g$ are more frequent closer to the ground. This is consistent with the results of Vickers and Mahrt (2003), who also found an increase of $\tau _g$ with height above ground. At VF0, a similar difference can be observed for the smallest bin, which is, however, not equally pronounced in other variables. Small $\tau _g$ are overall somewhat less frequent at SF1 and NF27_lvl2, with the exact distributions depending again on the variable. At SF1, wind speeds are generally higher than at the other sites during nighttime and at NF27, downslope winds occur regularly (Lehner et al. 2021), with strong vertical wind shear.

### Correlation Between the Gap Scale and Mean Flow Parameters

**Fig. 7:**
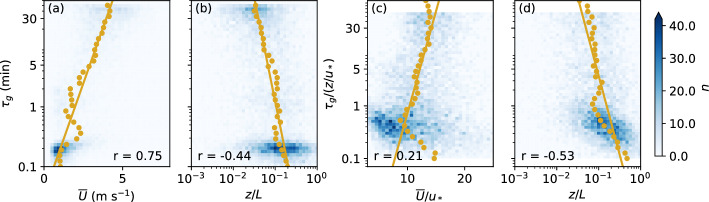
Distribution of $\tau _g$ identified from $D_{uu}$ using VK07 as a function of **a** mean wind speed and **b** stability at VF0_lvl1. (**c**, **d**) as (a, b) but $\tau _g$ and ${\overline{U}}$ are normalized by $z/u_*$ and $u_*$, respectively. The yellow lines show fits (Eq. 3) through the binned data (yellow dots) and *r* is the Spearman correlation coefficient

To determine an expression for a time-varying filter time, a relation needs to be found between the identified gap time scale $\tau _g$ discussed in the previous subsection and the local mean flow parameters. Figures 7a,b show an example of the relationship between $\tau _g$ and the mean wind speed ${\overline{U}}$ and between $\tau _g$ and the stability parameter *z*/*L*, respectively. Specifically, the example shows the results for VF0_lvl1 and $\tau _g$ identified from $D_{uu}$ using VK07. ${\overline{U}}$ and *z*/*L* are 60-min averages calculated from individual 30-min values. While there is significant scatter in the data, there is also a distinct correlation with both parameters, with higher wind speeds and lower *z*/*L* resulting in larger $\tau _g$ as expected. Similar relations can also be found for other combinations of variables, methods, types of (co)spectra, and stations (not shown). To fit a curve through the data, data points exceeding the mean fitting variables (${\overline{U}}$ or *z*/*L*) by at least 5 standard deviations were excluded. VK07 can yield values of $\tau _g$ larger than 60 min, that is, outside the range of the original (co)spectra, because it is based on an analytical form of a polynomial fit to the (co)spectra. To avoid using values of $\tau _g$ based on potentially unrealistic fits outside the range of the original (co)spectra, only $\tau _g$ smaller than 50 min were used for fitting. This ensures further that no values are included, which correspond to the last point of the (co)spectrum if identified with the method of VM03. The remaining $\tau _g$ were binned and median values of ${\overline{U}}$ or *z*/*L* calculated for each bin, indicated by the yellow dots in Fig. 7. An exponential curve of the form:

3 $$\begin{aligned} \tau _g = \exp (a x + b), \end{aligned}$$

was fitted through the binned values, where *a* and *b* are the fit parameters and $x={\overline{U}}$ or $x=\ln (z/L)$. Both ${\overline{U}}$ and *z*/*L* are local values from the same measurement level as the identified $\tau _g$. Only bins with at least 10 valid data points were included in the fit and no fit was determined if less than three bins with enough valid data were present.

**Fig. 8:**
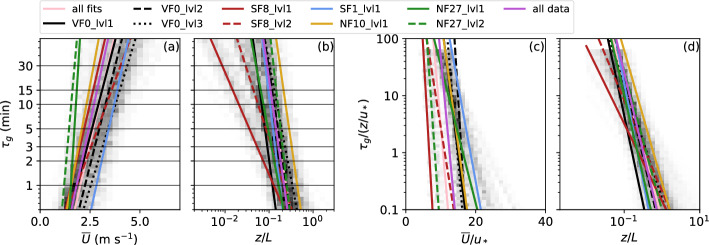
Frequency distribution of all fits (Eq. 3) with a *p* value less than 0.01 (gray shading) for $\tau _g$ as a function of **a** ${\overline{U}}$ and **b** *z*/*L* and normalized $\tau _g$ as a function of **c** normalized ${\overline{U}}$ and **d** *z*/*L*. The pink line corresponds to the median fit parameters *a* and *b* from all individual fits using different variables, types of (co)spectra, methods, and sites and other colored lines show the median fits for each of the different measurement sites. The purple line is based on fits of data from all locations

The same method was applied to all sites, variables, types of (co)spectra, and both methods of identifying $\tau _g$, yielding a range of different fits for both ${\overline{U}}$ and *z*/*L* (Fig. 8a, b). Only fits with a *p* value lower than 0.01 have been retained for the analysis and, in addition, fits based on spectra of *w* were removed because the identification of $\tau _g$ does not work well. Some fits based on data with large scatter also produced a slope with a sign opposite to expectation and to the majority of the fits, that is, a negative slope for ${\overline{U}}$ and a positive slope for *z*/*L*. These curves were particularly frequent for SF8 and were not included in the median fits shown in Fig. 8a, b either. While the remaining fits show some scatter, there are little systematic differences based on the variable and spectral method (not shown). A large part of the scatter is, however, a result of location so that median fits for each sonic anemometer differ from the medians over all fits and from each other. For example, individual fits for NF27 are clustered in the left part of Fig. 8a, which is partly a result of the overall low wind speeds observed at this site, while other sites are oftentimes influenced by the stronger valley winds (Lehner et al. 2021). Fits for SF8, in particular SF8_lvl1, on the other hand, extend further to near-neutral conditions (Fig. 8b). SF8 is located next to a concrete parking lot, where *z*/*L* remains weak during stable periods before sunset and stability weakens rapidly in the morning after sunrise.

In addition, fits were also calculated in a non-dimensional framework. The gap scale $\tau _g$ was normalized by $z/u_*$ and the wind speed ${\overline{U}}$ by $u_*$, where $u_*$ is the friction velocity, calculated over the same averaging period as ${\overline{U}}$. Since the goal of this work is to evaluate the performance of a time-varying filter time that can be relatively easily determined, normalization with more complex parameters, such as the integral time scale, were not considered. Scatter does not decrease compared to the non-normalized framework, neither for the correlation between $\tau _g$ and the mean flow parameters (Fig. 7) nor among the individual fits resulting from different (co)spectra and identification methods (Fig 8).

**Fig. 9:**
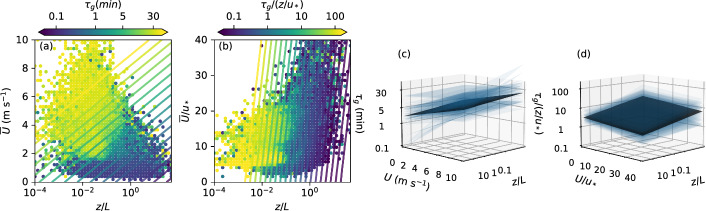
**a**, **b** Distributions of binned **a** $\tau _g$ and **b** normalized $\tau _g$ identified from $D_{uu}$ using VK07 as a function of *z*/*L* and **a** ${\overline{U}}$ and **b** $\overline{U/u_*}$ from all measurement locations. Color lines show the respective multi-variable fits (Eqs. 4, 5). **c**, **d** Comparisons of multi-variable fits from different methods and (co)spectra using data from all measurement locations in a **c** dimensional and **d** non-dimensional framework (transparent blue planes). The black planes show the respective median fits

To apply a multi-variable regression using both *z*/*L* and ${\overline{U}}$, the identified $\tau _g$ were first binned and only bin averages with at least three data points were used for the regression. Example distributions of $\tau _g$ in the two-dimensional parameter space are shown in Fig. 9a, b for both dimensional and non-dimensional data. Multi-variable regressions of the form:

4 $$\begin{aligned} \tau _g&= \exp \left( c \ln \frac{z}{L} + d {\overline{U}} + e\right) , \end{aligned}$$

5 $$\begin{aligned} \frac{\tau _g}{z/u_*}&= \exp \left( c \ln \frac{z}{L} + d\frac{{\overline{U}}}{u_*} +e \right) , \end{aligned}$$

where *c*, *d*, and *e* are the fitting coefficients, describe the general behavior of $\tau _g$, with an increase of $\tau _g$ with decreasing *z*/*L* and increasing ${\overline{U}}$ or decreasing ${\overline{U}}/{u_*}$. In the example shown in Fig. 9a, b for $D_{uu}$, the fit seems to somewhat overestimate $\tau _g$ using dimensional data for near-neutral conditions, with a better agreement for non-dimensional data. A comparison of all fits resulting from the different variables, types of (co)spectra, and both methods VM03 and VK07 are shown in Fig. 9c, d. Similar to the single-variable regressions (Eq. 3), only those curves with *p* values less than 0.01 for both fit parameters and with the expected and dominant slopes were included. In the next section, the determined regression curves are used to derive a time-varying filter time and the performance of the different fits is evaluated.

To evaluate the robustness of the fits to some degree, we recalculated them for individual subperiods of the total 2014–2020 period, specifically for the three subperiods 2014–2015, 2016–2017, and 2018–2019. The median fits (not shown) for each site are very close to those for the whole period shown in Fig. 8 for the dimensional fits, except for one outlier at SF1. It has to be kept in mind that shorter periods mean fewer data points, which is detrimental for the fitting algorithm, which fails more frequently or yields unreliable results. The variability is also somewhat larger for the non-dimensional site-specific fits, in particular for SF8 and NF27. The medians over all individual fits and the fits from all data (pink and purple lines in Fig. 8) vary, however, very little, using both dimensional and non-dimensional analysis, as do the multi-variable fits. We similarly calculated fits using 60-min averages of ${\overline{U}}$ and *z*/*L* calculated from individual 1-min values. The differences to the fits calculated from 30-min data are of a similar magnitude as the variations among the different subperiods (not shown) and have little impact on the predicted distributions of the filter time discussed in the next section.

## Impact of Filter Time

### Time-Varying Filter Time

In this section, we want to evaluate the performance of different constant filter time scales between $\tau _{fc}=30$ s and 30 min and of time-varying filter times based on the fits described above. The performance will be evaluated using two separate criteria that are designed to establish whether a given filter time removes non-turbulent motions effectively and whether the filter time is long enough to capture most of the turbulence spectrum. These criteria are described in detail in Sect. 5.1.2. To determine a time-varying filter time, $\tau _{fv}$, data were pre-processed with constant filter times $\tau _{fc}$ of 30 s, 1 min, 2 min, 3 min, 5 min, 10 min, 15 min, and 30 min. Using, for example, the 30-min averaged values of ${\overline{U}}$ together with the coefficients determined from the regression in Eq. 3, yields one value of $\tau _{fv}$ for each 30-min period, for which the closest value within the above list of filter times is selected. The data for this 30-min period are then extracted from the pre-processed datasets. All fluxes and turbulence statistics for $\tau _{fv} < 30$ min are averaged over a 30-min period, that is, if $\tau _{fv}=3$ min for a given 30-min period, the ten 3-min periods are averaged to give a single value. This approach is easier to implement than actually processing the data with a time-varying filter time, in particular with many existing eddy-covariance software packages.

#### Predicted Distributions of $\tau _{fv}$

**Fig. 10:**
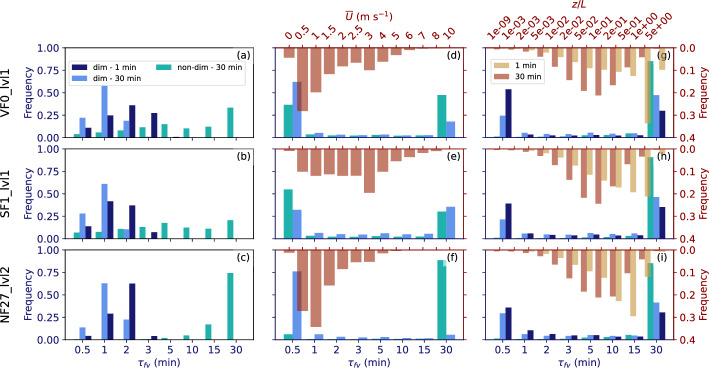
Distributions of $\tau _{fv}$ for all stable 30-min periods at VF0_lvl1 (top row), SF1_lvl1 (middle row), and NF27_lvl2 (bottom row). **a**–**c** $\tau _{fv}$ based on dimensional (light and dark blue bars) and non-dimensional (turquoise bars) multi-variable regressions; **d**–**i** $\tau _{fv}$ based on dimensional and non-dimensional single-variable regressions using **d**–**f** ${\overline{U}}$ and **g**–**i** *z*/*L* as input parameters. Input parameters ${\overline{U}}$ and *z*/*L* are based on $\tau _{fc}=30$-min (light blue) and $\tau _{fc}=1$-min (dark blue) averages. In addition, **d**–**i** show the distributions of **d**–**f** the $\tau _{fc}=30$-min mean wind speed (brown bars) and **g**–**i** the $\tau _{fc}=30$-min (brown bars) and 1-min (yellow bars) stability parameter (top *x* axes and right *y* axes). Distributions of $\tau _{fc}=1$-min mean wind speed are not included in **d**–**f** because the scalar-averaged wind speed does not depend on $\tau _{fc}$ and the distribution is thus identical to that using $\tau _{fc}=30$-min

The performance of $\tau _{fv}$ based on different regressions is evaluated, specifically, the medians of all single-variable regressions shown in Fig. 8 (pink lines), as well as the median multi-variable fits (Fig. 9) from both dimensional and non-dimensional analysis. Since the regressions show significant differences among the locations, site-specific regression curves (color lines in Fig. 8) are tested as well. Distributions of $\tau _{fv}$ from different regression lines are shown in Fig. 10 together with the distributions of the $\tau _{fc}=1$-min and 30-min mean wind speed and *z*/*L*, with the 1-min data averaged to 30 min. While the filter time used to calculate *z*/*L* has relatively little impact on the regression curves themselves, it has a non-negligible impact on the distribution of $\tau _{fv}$ resulting from *z*/*L*. In connection with the regression lines in Fig. 8, the distributions of ${\overline{U}}$ and *z*/*L* help to explain the final distributions of $\tau _{fv}$.

Observed wind speeds during stable conditions are overall low (Fig. 10d–f), particularly at the valley floor, with strongly skewed distributions. At VF0_lvl1, ${\overline{U}}<3$ m s$^{-1}$ for more than 80% of all 30-min periods and ${\overline{U}}<1$ m s$^{-1}$ for more than 30%. The corresponding $\tau _{fv}\left( {\overline{U}}\right)$ resulting from the curve shown in Fig. 8 are thus equally low, yielding $\tau _{fv}=30$ s in more than 60% of the time. This result seems to differ from the distributions of the gap scale $\tau _g$ shown in Fig. 6, which indicate a much larger number of values above 1 min and even above 20 min. This may point to an imperfect representation of the data, which scatter strongly, by the respective regression curves. A cluster of high $\tau _g$ can, for example, be seen in Fig. 7a for ${\overline{U}}$ between 2 and 6 m s$^{-1}$. In addition, the distributions of wind speed for the periods, for which the gap scale identification is successful are partly shifted to slightly higher values, in particular at VF0 and SF1 (not shown). Using site-specific fits does not change the distributions of $\tau _{fv}$ much, except for NF27, where the distribution is shifted more towards 30 min (not shown). The distribution changes, however, at all sites when using the regressions based on non-dimensional parameters for both site-specific and general fits (Fig. 10d–f). The difference is, however, mainly in shifting values from $\tau _{fv}=30$ s to $\tau _{fv}=30$ min.

Using *z*/*L* as the independent variable instead of ${\overline{U}}$ leads to an overall smaller number of periods with $\tau _{fv}=30$ s and slightly higher numbers in all other classes (Fig. 10g–i) when *z*/*L* is based on $\tau _{fc}=30$ min. The distributions are similar to those of $\tau _{fv}\left( {\overline{U}}\right)$ when using $\tau _{fc}=1$ min since more periods are classified as strongly stable (yellow bars), leading to lower $\tau _{fv}$ based on the curves shown in Fig. 8b. Similar to ${\overline{U}}$, the function based on non-dimensional parameters yields $\tau _{fv}=30$ min most of the time.

Using a multi-variable regression and including both *z*/*L* and ${\overline{U}}$ as predictors yields completely different distributions (Fig. 10a–c). Instead of identifying $\tau _{fv}=30$ s as the appropriate filter time for most of the periods, the distribution shifts to $\tau _{fv}=0.5-3$ min at all sites, with the exact distribution depending on the site. $\tau _{fv}$ depends also on whether *z*/*L* used in Eq. 4 to calculate $\tau _{fv}$ is based on 30-min or 1-min averaged fluxes. Using $\tau _{fc}=30$ min input data, the distribution is shifted to lower values of $\tau _{fv}$. For the non-dimensional fit, the distributions are, however, similar to those resulting from the single-variable fits. The filter time thus depends strongly on the type of fit (multi-variable vs single-variable) and on the frequency distribution of the independent variables used to calculate $\tau _{fv}$.

#### Performance Assessment

The goal of using a time-varying filter time is to ideally separate turbulent and non-turbulent motions better than with a constant filter time. To evaluate the performance of $\tau _{fv}$ and different $\tau _{fc}$, we thus need to determine whether the selected filter time for each 30-min period is (i) short enough to remove most non-turbulent motions and (ii) long enough to capture most of the turbulence spectrum. To estimate whether $\tau _f$ is short enough, the scalar-averaged mean wind speed ${\overline{U}}$ is compared to the vector-averaged mean wind speed ${\overline{u}}$, where ${\overline{u}}$ corresponds to the wind speed in the mean wind direction of the averaging period. It is quite common to define ${\overline{u}}$ as the mean wind speed when processing eddy-covariance data, for example in the frequently used EddyPro Software (LI-COR Biosciences 2021). The vector-averaged mean wind speed can, however, be significantly lower than the scalar-averaged wind speed, depending on the variability of the wind direction (Clive 2008). In the presence of oscillatory motions in the horizontal wind direction with a time scale shorter than $\tau _f$, such as, for example, meandering (Mortarini et al. 2016, 2019), ${\overline{U}}$ and ${\overline{u}}$ can thus differ strongly. If $\tau _f$, on the other hand, is short enough to remove these large-scale motions, ${\overline{u}}$ should be close to ${\overline{U}}$ and the difference $\varDelta u = |{\overline{U}} - {\overline{u}}|$ can thus be used to quantify the performance of $\tau _f$. To estimate whether $\tau _f$ is at the same time long enough to capture most of the turbulence spectrum, the spectra of the vertical velocity are used, which show comparatively little impact of non-turbulent motions at larger scales (Fig. 2). The spectra decrease continuously for scales larger than the turbulence peak, so that the variances calculated with increasing $\tau _f$ will converge. A reference $\tau _{f,ref}=30$ min is defined to represent the total turbulent variance $\overline{w'^2}_{ref}$ and the difference $\varDelta \overline{w'^2} = |\overline{w'^2}_{ref} - \overline{w'^2}_{\tau _f}|$ represents the underestimation of the total variance when using $\tau _f < 30$ min and is used to quantify how much of the turbulence spectrum is missed.

**Fig. 11:**
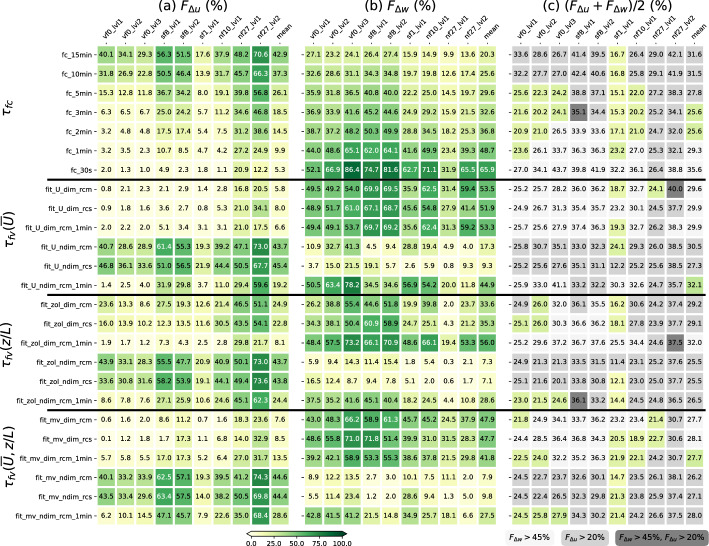
Frequencies **a** $F_{\varDelta u}$ and **b** $F_{\varDelta w}$ and **c** the mean of $F_{\varDelta u}$ and $F_{\varDelta w}$ for different constant filter times (top rows) and for $\tau _{fv}$ from different regression curves (bottom rows), where *U* and *zol* indicate the fitting variables ${\overline{U}}$ and *z*/*L*, respectively; *mv* a multi-variable fit; *rcs* and *rcm* the site-specific and median over all regression curves, respectively; *dim* and *ndim* whether the fits are based on the dimensional or non-dimensional analysis; and *1min* that ${\overline{U}}$ and *z*/*L* are based on $\tau _{fc}=1$-min data instead of 30-min data. The individual columns in the subfigures show the respective frequency for each of the sensor locations and the last column (*mean*) is the respective mean of all sensor locations

Figure 11a shows the frequency $F_{\varDelta u}$ of $\varDelta u/{\overline{U}} > 10$% for different constant filter times $\tau _{fc}$ and different regression curves used to determine $\tau _{fv}$. For a constant $\tau _{fc}$, $F_{\varDelta u}$ increases with increasing $\tau _{fc}$, reaching, for example, values of about 40% for $\tau _{fc}=15$ min at VF0_lvl1. This means, using $\tau _{fc}=15$ min, non-turbulent motions are not completely removed in 40% of all cases. $F_{\varDelta u}$ differs, however, from site to site. At NF27_lvl2, $\varDelta u$ exceeds 10% in more than 70% of all cases using $\tau _{fc}=15$ min, whereas at SF1, the number is below 20%. While $F_{\varDelta u}$ decreases rapidly with decreasing $\tau _{fc}$ for most sites, it remains high at NF27 for all $\tau _{fc}$. Even with $\tau _{fc}=30$ s, $F_{\varDelta u}$ is about 20% at NF27_lvl1. This suggests that at NF27, wind direction is strongly variable even at small time scales. These motions overlap with the turbulence scale and are thus difficult to remove with a traditional block averaging filter.

$F_{\varDelta w}$, that is, the frequency of $\varDelta \overline{w'^2}/\overline{w'^2}_{ref}>10$% shows the opposite trend to $F_{\varDelta u}$, that is, it generally increases with decreasing $\tau _{fc}$ (Fig. 11b). Using $\tau _{fc}=30$ s, the total variance is underestimated by more than 10% in more than 60% of the cases at most of the sensor locations, which means that $\tau _{fc}=30$ s is generally too short to capture all of the turbulence. The most pronounced outlier is NF27_lvl1 at 1.5 m a.g.l., where this is only true about 30% of the time. The dependence of $F_{\varDelta w}$ on $\tau _{fc}$ is, however, overall not as large as that of $F_{\varDelta u}$. Even with $\tau _{fc}=15$ min, $F_{\varDelta w}$ remains above or close to 15% at all locations, except for NF27_lvl2.

For $\tau _{fv}$, the results depend strongly on the used regression. High values of $F_{\varDelta u}$ together with relatively low values of $F_{\varDelta w}$ are found for fits based on a non-dimensional analysis when using $\tau _{fc}=30$ min data to determine $\tau _{fv}$, independent of the fit variable. This is a result of the high number of $\tau _{fv}=30$ min intervals identified by these fits (Fig. 10). $F_{\varDelta u}$ decreases when using $\tau _{fc}=1$-min data to determine $\tau _{fv}$ as a result of the high number of periods with $\tau _{fv}=30$ s (Fig. 10). The dimensional fits based on ${\overline{U}}$ yield a large number of $\tau _{fv}=30$ s (Fig. 10); $F_{\varDelta u}$ is thus low and $F_{\varDelta w}$ correspondingly high, similar to $\tau _{fc}=30$ s. The multi-variable dimensional fit resulted in completely different distributions of $\tau _{fv}$, with the highest numbers between $\tau _{fv}=30$ s and 3 min (Fig. 10). The resulting $F_{\varDelta u}$ and $F_{\varDelta w}$ based on 30-min and 1-min averaged values are thus similar to those for $\tau _{fc}=0.5-1$ min and $\tau _{fc}=2-3$ min, respectively.

If we want to identify a filter time that performs best, both scores $F_{\varDelta u}$ and $F_{\varDelta w}$ need to be combined to determine the $\tau _f$ that removes the non-turbulent motions best while, at the same time, captures most of the turbulent motions. Figure 10 shows that all fits yield a distribution of $\tau _{fv}$ with one or two dominant peaks, resulting in similar scores than the respective $\tau _{fc}$. None of the tested fits stands out with low values of both $F_{\varDelta u}$ and $F_{\varDelta w}$, indicating a perfect performance. It can also be seen from Fig. 11 that $F_{\varDelta u}$ depends more strongly on $\tau _f$ than $F_{\varDelta w}$. Keeping $F_{\varDelta w}$ below 45% and $F_{\varDelta u}$ below 20% (green squares in Fig. 11c), $\tau _{fc}=2-3$ min emerge as the overall best choices based on the mean values over all sites (Fig. 11c). Among the different time-varying filter times, the multi-variable dimensional fit using $\tau _{fc}=1$ min as input data yields the best result, but slightly worse than $\tau _{fc}=2-3$ min.

For individual locations, results, may differ, however. Similar results are found at VF0 and NF10. At VF0, the mean of $F_{\varDelta u}$ and $F_{\varDelta w}$ reaches its minimum for larger values of $\tau _{fc}$ at the top two measurement levels, in agreement with the previous finding that the gap scale increases with height. It has to be mentioned, however, that vertical velocity spectra at VF0 are most strongly impacted by non-turbulent motions in the low-frequency range compared to other locations (Fig. 2), likely related to the frequently occurring oscillatory motions in the very stable and nearly quiescent layer above the valley floor. This impact in the spectra means, however, that $F_{\varDelta w}$ is potentially even overestimated. At SF8 and NF27_lvl2, on the other hand, none of the tested $\tau _{fv}$ and $\tau _{fc}$ yields $F_{\varDelta u} < 20$% and $F_{\varDelta w} < 45$%. At NF27, this is largely due to high values of $F_{\varDelta u}$, suggesting that non-turbulent motions occur within the katabatic flows at time scales lower than 1 min. Oscillations have been observed frequently in downslope and other drainage flows (Zardi and Whiteman 2012). SF8, on the other hand, shows high values of both $F_{\varDelta u}$ and $F_{\varDelta w}$, with $F_{\varDelta w}$ increasing faster with increasing $\tau _{fc}$ than at other sites. As mentioned before, near-neutral conditions occur more frequently at SF8, with typically higher spectral densities in the inertial subrange and near the turbulence peak (not shown), thus likely causing a fast underestimation of the total turbulence spectrum for lower $\tau _{fc}$. At both SF8 and NF27, the lowest mean values of $F_{\varDelta u}$ and $F_{\varDelta w}$ for constant filter times are, however, equally reached for $\tau _{fc}=2$ min and the dimensional, multi-variable fit yields one of the lowest values among all tested fits for NF27, but not SF8. The lowest values of $F_{\varDelta u}$ and $F_{\varDelta w}$ occur generally at SF1, with equally low mean values for $\tau _{fc}=2$–15 min, which are also much lower than at the other sites. The location of SF1 on an almost flat plateau above the valley floor is special in that it is typically characterized by higher wind speeds during nighttime than the other sites (Lehner et al. 2021), but no physical explanation for the difference in $F_{\varDelta w}$ and $F_{\varDelta u}$ can be brought forward at the moment.

### Impact of the Filter Time on Turbulence Statistics

**Fig. 12:**
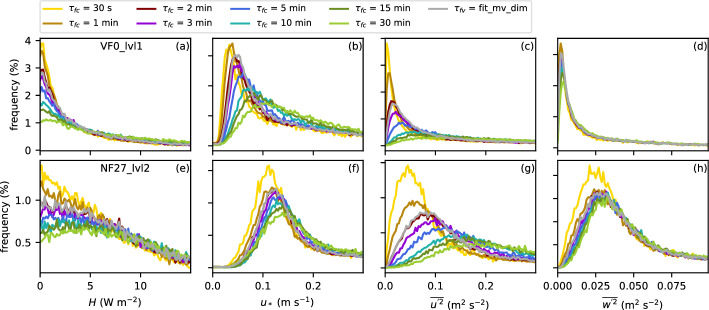
Distributions of **a**, **e** the sensible heat flux, **b**, **f** the friction velocity, **c**, **g** the variance of the streamwise wind component, and **d**, **h** the variance of the vertical wind component at **a**–**d** VF0_lvl1 and **e**–**h** NF27_lvl2 for different $\tau _{fc}$ (color lines) and $\tau _{fv}$ based on the dimensional multi-variable fit using $\tau _{fc}=1$ min input data in Eq. (4) (gray lines). Note the difference in the scale of the vertical axes in (**a**–**d**) and (**e**–**h**)

The final aspect to be addressed is the impact of the filter time $\tau _f$ on the turbulent fluxes and other turbulence statistics, that is, the question of how important the selection of an appropriate filter time actually is. Figure 12 shows distributions of the sensible heat flux, the friction velocity, and the variances of the streamwise and the vertical wind component at VF0_lvl1 and NF27_lvl2 for all 30-min stable periods using different $\tau _{fc}$ and $\tau _{fv}$ based on the dimensional multi-variable fit with $\tau _{fc}=1$-min input data. As expected, the distributions are generally shifted towards smaller fluxes and variances for smaller $\tau _{fc}$. The exact changes differ, however, among sites and also variables. At VF0_lvl1, $\tau _{fc}=30$ s yields very similar sensible heat and momentum fluxes as $\tau _{fc}=1$ min, whereas at NF27_lvl2 the difference is much larger. The particularly large change between $\tau _{fc}=1$ min and 30 s at NF27_lvl2 is also obvious in the variances of the two wind components. The turbulent fluxes and variances are overall much smaller at VF0_lvl1 than at NF27_lvl2, independent of $\tau _f$. While VF0 is located at the valley bottom within a very quiescent and stable near-surface layer during nighttime, NF27 is located on a slope, where katabatic winds with strong vertical wind shear occur regularly. Turbulence intensities are thus generally higher at NF27 and the strong difference between $\tau _{fc}=30$ s and other $\tau _{fc}$ and $\tau _{fv}$ at NF27 suggests, that 30 s is generally too short to capture the full turbulence spectrum.

**Fig. 13:**
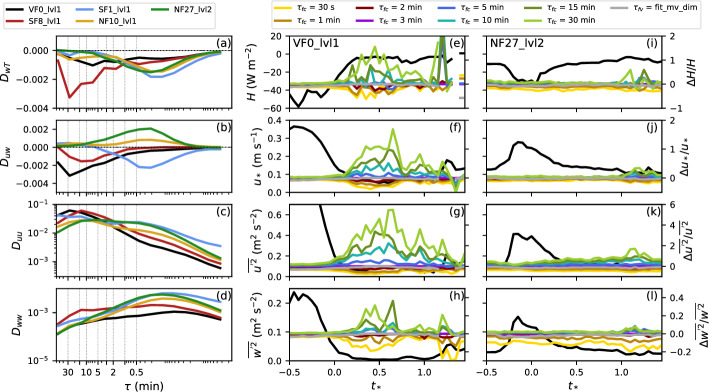
**a**–**d** Median MRD (co)spectra from periods between sunset and sunrise of 94 undisturbed nights. Vertical dotted lines indicate the used $\tau _{fc}$ between 30 s and 30 min. **e**–**l** Median time series of **e**, **i** the sensible heat flux, **f**, **j** the friction velocity, **g**, **k** the variance of the streamwise wind component, and **h**, **l** the variance of the vertical wind component at VF0_lvl1 and NF27_lvl2 during undisturbed nights using $\tau _{fc}=3$ min (black lines, left axes) and the respective relative deviations using different $\tau _{fc}$ (color lines, right axes) and $\tau _{fv}$ based on the dimensional multi-variable fit using $\tau _{fc}=1$ min input data in Eq. (4) (gray lines, right axes). The time axes are normalized by the length of the night between sunset and sunrise, with $t_*=0$ and $t_*=1$ marking sunset and sunrise, respectively

The impact of $\tau _f$ is generally smaller for the variance of the vertical wind component, except for $\tau _{fc}=30$ s at NF27_lvl2. This is consistent with the previous finding that the spectra of *w* decrease monotonously for $\tau$ larger than the turbulent peak, so that the variance converges with increasing $\tau _{fc}$. This can also be seen during individual nights. While Fig. 12 includes data for all stable conditions, Fig. 13 shows median time series of the same variables using $\tau _{fc}=3$ min at VF0_lvl1 and NF27_lvl2 and the relative deviations using different $\tau _f$ for 94 synoptically undisturbed and clear-sky conditions identified analogously to Lehner et al. (2019). The choice of $\tau _f$ has a much larger influence on the results at VF0_lvl1 than at NF27_lvl2, which can be at least partly explained by the overall lower values of turbulent fluxes and variances, for example, the friction velocity at VF0, thus resulting in a larger relative difference. The variability among the different $\tau _f$ is also smaller for $\overline{w'^2}$ during these nights compared to the other variables. For $\overline{u'^2}$, the relative difference can reach values up to 500% at VF0_lvl1 and even at NF27_lvl2 it reaches values of up to 200%. In particular, $\tau _{fc}=10-30$ min differ strongly from the other curves. These may be considered long filter times for stable conditions, which are thus rarely used. However, even for common choices of $\tau _{fc}=1-5$ min, results can vary by up to about 50%.

The corresponding MRD spectra show large contributions from submeso motions in the low-frequency range, which are particularly large at VF0 and SF8 close to the valley floor (Fig. 13c). During undisturbed conditions, the layer directly above the valley floor is typically strongly stratified with very low wind speeds, where oscillations are observed frequently. At VF0, the submeso contributions are much smaller in the vertical velocity spectra (Fig. 13d), suggesting meandering motions. These motions further contribute to the larger sensitivity of the fluxes and variances to the filter time at VF0 than at NF27, NF10, and SF1. At SF8, low-frequency peaks are not only visible in the horizontal velocity spectrum and the momentum cospectrum, but also the vertical velocity spectrum and the heat flux cospectrum (Fig. 13a–d), which indicates that these contributions have a different origin than horizontal meandering. They also lead to a larger sensitivity of the sensible heat flux to the filter time, in particular for filter times shorter than 30 min (not shown).

Since the dimensional multi-variable fit yields mostly values of $\tau _{fv}=$1–3 min, the corresponding distributions are generally similar to the ones resulting from $\tau _{fc}=$2–3 min (Fig. 12). In particular at NF27, where $\tau _{fv}$ has a pronounced peak at 2 min (Fig. 10), the distributions of the turbulent fluxes using $\tau _{fv}$ are almost identical to $\tau _{fc}=2$ min. The example time series in Fig. 13 show, however, an overall relatively small dependency of *H* on the filter time at NF27_lvl2.

## Summary and Discussion

(Co)spectra from five eddy-covariance stations in the highly complex terrain of the Inn Valley, Austria, were analyzed for stable conditions. Relationships could be found between the energy gap time scale $\tau _g$ identified from the (co)spectra and the mean wind speed ${\overline{U}}$ and between $\tau _g$ and the stability parameter *z*/*L*. Using these relationships, a comparatively easy-to-use method of processing the turbulence data with a time-varying filter time $\tau _{fv}$ was presented. The data are pre-processed with a range of different constant filter times between 0.5 and 30 min. The identified regression curves are used to predict $\tau _{fv}$ for each 30-min averaging interval based on pre-processed values of ${\overline{U}}$ and *z*/*L* and the respective data are then extracted from the pre-processed datasets. While this approach results only in a discrete number of different values for $\tau _{fv}$, in this case specifically eight values (0.5, 1, 2, 3, 5, 10, 15, and 30 min), it is relatively easy to use with many existing eddy-covariance processing software packages, which allow only a single constant filter time to be specified for the entire dataset. At the same time, the pre-processed datasets are also used to determine the mean flow parameters to predict $\tau _{fv}$.

In this study, we determined the gap scale $\tau _g$ from (co)spectra of different variables, including the spectra of temperature, humidity, and the three wind components and the cospectra of the momentum, heat, and moisture fluxes in all three directions. In addition, we applied different methods to (i) calculate the (co)spectra and (ii) determine $\tau _g$. Following an approach proposed by Vickers and Mahrt (2003), $\tau _g$ was determined from (co)spectra calculated using the multi-resolution flux decomposition method, identifying extrema in the (co)spectral curves. The same approach was also applied to Fourier (co)spectra. A second method was used to fit a polynomial function to the (co)spectra, which allows an analytical determination of the curve’s extrema following Voronovich and Kiely (2007). The results showed that the distributions of the identified gap scales differ more strongly among different types of (co)spectra and methods at a single site than from site to site using the same (co)spectrum. The range of $\tau _g$ identified for a single site shows that the results depend strongly on the choice of method and variable and highlights the overall challenge in identifying an appropriate filter time from spectral analysis. Part of these differences result from, for example, the stronger damping of vertical motions close to the ground compared to horizontal motions, but also the impact of non-turbulent motions at low frequencies, which may not affect all (co)spectra equally. For example, meandering motions will have a stronger impact on horizontal than on vertical motions, while linear gravity waves transport momentum, but not heat. This sensitivity to the selected variables is important to keep in mind when comparing gap scales from the different studies.

The large variability of $\tau _g$ and its strong dependence on the variables, types of (co)spectra, and methods used for gap identification also raise the question whether it is even possible to determine the optimal $\tau _g$ using spectral analysis. To include this uncertainty, an ensemble approach was used for further analysis. Linear regressions were computed between the mean flow parameters and all $\tau _g$ identified from the different methods, types of (co)spectra, and variables, using both single-variable and multi-variable regressions. Median single-variable and multi-variable fitting functions were determined subsequently from this ensemble of fit parameters. Despite the large variability of the gap scales identified from different (co)spectra and using different methods, the fits with the mean flow parameters ${\overline{U}}$ and *z*/*L* showed very consistent trends. The resulting median regression curves used to predict time-varying filter times are thus robust enough to evaluate the impact of using a time-varying filter time compared to using a constant filter time.

The performance of the time-varying filter time resulting from single-variable regressions with ${\overline{U}}$ and *z*/*L* and from multi-variable regressions was evaluated by estimating how well the respective $\tau _{fv}$ removes non-turbulent motions and, at the same time, captures most of the turbulence spectrum. The criteria to evaluate the performance of both $\tau _{fv}$ and different constant filter times were designed to determine how often more than 10% of the turbulence spectrum are missed and how often non-turbulent motions clearly affect the results. The criteria are thus implicitly based on the assumption of a distinct energy gap so that the turbulent and non-turbulent motions can be clearly separated. Particularly under stable conditions, the spectral ranges of turbulence and non-turbulent submeso motions may, however, overlap. This means that it may not be possible to remove non-turbulent motions and capture the full turbulence spectrum under these conditions using simple block averaging. Other filter types may be needed to clearly separate turbulent and non-turbulent motions, but it is also possible that such filters do not exist for all conditions. In addition, the criterion to determine how much of the turbulence spectrum is missed, is based on the assumption that the vertical-velocity spectra are not affected by non-turbulent motions. While the median spectra show a continuous decrease at scales larger than the turbulence peak in contrast to the horizontal-velocity spectra, it cannot be excluded that, for example, gravity waves occur occasionally, which will also impact the vertical velocity.

When comparing the performance of time-varying filter times with that of constant filter times, none of the time-varying filter times performed better than a well chosen constant filter time. At the five i-Box sites, constant values of $\tau _{fc}=2-3$ min yielded the best overall performance, with similar results using a time-varying filter time based on a multi-variable regression. The latter performed very similarly to $\tau _{fc}=2$ min since the distribution of the predicted $\tau _{fv}$ has a prominent peak at 1–3 min. This could indicate that $\tau _{fv}$ does not vary strongly during stable conditions at these sites and that a constant filter time thus yields good results. To confirm this hypothesis, it would, however, be necessary to extend the analysis to other sites and conditions to determine (i) whether different distributions of $\tau _{fv}$ are found and (ii) whether a time-varying filter time can outperform a constant filter time if $\tau _{fv}$ is more evenly distributed. Performing the same analysis based on a subset of (co)spectra with mainly stationary conditions did not have a significant effect on the results.

## Footnotes

1 The datasets are available on zenodo (Lehner 2023a, b, c, d, e, f, g, h).
